# Annual Report of the Medical Society of the Canton of Geneva

**Published:** 1846-01

**Authors:** 


Art. II.—*Rapport Annuel sur les Travaux de la Société Médicale du Canton de Genève. Pour l'année* 1843. Par le Docteur Marc d'Espine, President de la Société.

*Annual Report of the Medical Society of the Canton of Geneva.* By Dr. Marc d'Espine.

We select from this report one or two points of interest.

M. Morin read a paper to the society, on certain preparations from sweet and bitter almonds. *Amygdalin,* a proximate principle, consisting of oxygen, hydrogen, carbon, and azote, is found only in the bitter almond, while *emulsion* is found both in the bitter and sweet almond. All mixtures of *amygdalin* and *emulsion* in water give rise to the immediate formation of the essence of bitter almonds and prussic acid. The therapeutic properties of laurel-water depend upon its containing a quantity, unfortunately variable, of prussic acid. Liebig has proposed to substitute for the laurel-water an ounce of almond emulsion, mixed with seventeen grains of amygdalin. M. Morin finds this proportion of amygdalin too strong, and proposes only twelve grains of that substance, and asserts that the mixture will yield five grains and a half of the essential oil of bitter almonds, and five grains and a half of medicinal prussic acid, which is equivalent to two-thirds of a grain of anhydrous prussic acid.

Doctor Mayor adds another to the list of successful cases of Cæsarian operation. The female was forty years of age, and pregnant of her first child. Her delivery was rendered impossible by a fibrous tumour of the uterus, which filled the true pelvis. Both mother and child are now in perfect health.

